# Urinary biomarkers for diagnosing acute kidney injury in sepsis in the emergency department

**DOI:** 10.1016/j.heliyon.2024.e41252

**Published:** 2024-12-14

**Authors:** Sumin Baek, Inwon Park, Seonghye Kim, Young Woo Um, Hee Eun Kim, Kyunghoon Lee, Jae Hyuk Lee, You Hwan Jo

**Affiliations:** a Department of Emergency Medicine, Seoul National University Bundang Hospital (SNUBH), Seongnam-si, South Korea; b Department of Emergency Medicine, Seoul National University College of Medicine, Seoul, South Korea; c Disaster Medicine Research Center, Seoul National University Medical Research Center, Seoul, South Korea; d Department of Laboratory Medicine, Seoul National University Bundang Hospital (SNUBH), Seongnam-si, South Korea; e Department of Laboratory Medicine, Seoul National University College of Medicine, Seoul, South Korea

**Keywords:** Sepsis, Emergency service, Hospital, Acute kidney injury, Biomarkers, Tissue inhibitor of Metalloproteinase-2, Insulin-like growth factor binding proteins

## Abstract

**Background:**

Development of acute kidney injury (AKI) in patients with sepsis is associated with increased mortality, highlighting the importance of early detection and management. However, baseline creatinine or urine output measurements are required for AKI diagnosis, which can be challenging in emergency departments (EDs). We aimed to evaluate the association between urinary biomarkers and the AKI diagnosis or 30-day survival status in patients with sepsis in the ED.

**Methods:**

This prospective observational study enrolled patients from a single ED. We enrolled adult patients presenting to the ED with symptoms suggestive of infection and an initial quick sequential organ failure assessment score ≥2. Initial urine samples were collected, and urinary biomarkers (dickkopf-3, soluble triggering receptor expressed on myeloid cells-1, kidney injury molecule-1, neutrophil gelatinase-associated lipocalin (NGAL), and tissue inhibitor of metalloproteinases-2 (TIMP-2), and insulin-like growth factor binding protein-7 (IGFBP-7), and TIMP-2 × IGFBP-7) were analyzed using an enzyme-linked immunosorbent assay kit. Multivariable logistic regression models were used to evaluate biomarker performance.

**Results:**

Of 84 patients, 63 (75.0 %) were diagnosed with AKI and 16 (19.0 %) died within 30 days. None of the urinary biomarkers demonstrated significant differences between the survivors and non-survivors. NGAL (p = 0.014) and TIMP-2 × IGFBP-7 (p = 0.027) levels were different between the AKI and non-AKI groups. The multivariable logistic regression model suggested a higher area under the receiver operating characteristic curve for models, including TIMP-2 × IGFBP-7 (from 0.853 to 0.889, p = 0.018).

**Conclusion:**

None of the urinary biomarkers in the initial urine sample demonstrated an independent association with AKI diagnosis or 30-day survival status in patients with sepsis presenting to the ED. Further studies with larger population are necessary to confirm its clinical utility and explore its role.

## 1 Introduction

Sepsis is defined as fatal organ dysfunction caused by an uncontrolled host response to infection [1,2]. The kidneys are one of the predominantly affected organs in sepsis, suggesting a strong association between sepsis-associated acute kidney injury (AKI) and mortality [3,4]. The early and rapid detection of AKI in patients with sepsis, followed by appropriate treatment and injury prevention, is key to reducing high mortality in patients with sepsis [[4], [5], [6], [7], [8]].

AKI diagnosis and staging are based on increased serum creatinine relative to baseline and/or decreased urine output [9]. The pathophysiology of sepsis-associated AKI is distinct from that of other AKI causes; nonetheless, all AKI cases are diagnosed based on serum creatinine and urine output [9]. Furthermore, the baseline serum creatinine level is unknown, and urine output measurement is time-consuming and challenging in emergency departments (EDs), where the majority of patients with sepsis receive initial management [10]. Consensus methods for establishing pre-AKI baseline serum creatinine levels in the absence of previous measurements are unavailable [11]. Moreover, serum creatinine changes are often reflected later in AKI because of the renal reserve and kinetics of serum creatinine [6,12,13].

To overcome these limitations, novel biomarkers have been identified for diagnosing sepsis-associated AKI. Urine samples have been highlighted for their non-invasiveness and high specificity for kidney damage. Researchers have investigated several promising biomarkers, including dickkopf-3 (DKK-3) [14], soluble triggering receptor expressed on myeloid cells-1 (sTREM-1) [15], kidney injury molecule-1 (KIM-1) [16], neutrophil gelatinase-associated lipocalin (NGAL) [17], and tissue inhibitor of metalloproteinases-2 × insulin-like growth factor binding protein-7 (TIMP-2 × IGFBP-7) [18], and have supported their potential utility in prognostication and AKI staging for patients with sepsis. However, these studies were limited by insufficient direct comparisons among potential biomarkers in a single sepsis cohort. Moreover, the population consisted of patients admitted to the intensive care unit (ICU) instead of the ED [19]. It is unclear whether biomarkers in the initial urine collected upon arrival to the ED can serve as reliable biomarkers for AKI [20].

Therefore, we aimed to collect urine samples prospectively from patients presenting to the ED with symptoms suggestive of sepsis and to evaluate the correlation between potential biomarkers, AKI diagnosis, and patient survival.

## 2 Methods

### 2.1 Study design and population

We performed a prospective, observational study. Patient enrollment was conducted at a single ED of a university-affiliated hospital from January to May 2021. Patients who visited the ED with symptoms suggesting infection with an initial quick sequential organ failure assessment score (qSOFA) ≥2 points were enrolled [1]. The exclusion criteria were as follows: age <18 years, pregnancy, patients with anuria already diagnosed with end-stage renal disease on renal replacement therapy, and refusal to enroll. After enrollment, patients who did not meet the sepsis-3 definition were excluded [1].

### 2.2 Data collection and processing

To measure the urinary biomarkers, we used initial urine samples of the enrolled patients. The levels of urinary biomarkers (DKK-3, KIM-1, NGAL, sTREM-1, TIMP-2, and IGFBP-7) were measured using an enzyme-linked immunosorbent assay kit (DY1118, DKM100, DLCN20, DTRM10C, DTM200, DY1334, R&D Systems, Inc. Minneapolis, MN). As concentrations of urinary biomarkers of AKI are influenced by variation in urinary concentration, biomarkers were normalized to urinary creatinine [21,22].

Demographic information, comorbidities, initial hemodynamic measurements of the blood pressure, heart rate, respiratory rate, body temperature, and possible or confirmed primary site of infection were collected. Laboratory findings, such as blood gas analysis, complete blood count, C-reactive protein level, and serum chemistry, were collected. Mortality, the initiation of mechanical ventilation, the use of vasopressors, and application of renal replacement therapy (RRT) were collected as the outcome variables. The SOFA score was determined using parameters assessed with initial laboratory findings and vasopressor demands within a 6-h window after admission to the ED.

AKI was defined according to the serum creatinine category outlined by the Kidney Disease: Improving Global Outcomes criteria [9] as follows:

1) A 1.5- to 1.9-fold increase from the baseline value, or
2) An increase ≥0.3 mg/dL (≥26.5 μmol/L)
3) Urine output (<0.5 mL/kg/h for 6–12 h).

AKI diagnosis in the ED was established by comparing the baseline serum creatinine level with the first serum creatinine level upon initial presentation to the ED. Baseline creatinine was determined as the median value of creatinine measurements obtained during outpatient visits within a range of 365 to 8 days before ED visit [23,24]. In absence of a baseline value, we used the lowest inpatient serum creatinine level or urine output during the 48 h after enrollment. AKI development was defined by comparing the maximum value of creatinine within a 48-h period with the initial creatinine measurement or the volume of urine output.

The primary outcome was AKI development within 48 h of initial presentation at the ED. The secondary outcome was 30-day mortality after admission to the ED. Upon discharging the patients before the 30-day period, we performed a telephone follow-up to collect survival data.

### 2.3 Statistical analysis

The Shapiro–Wilk test was conducted to determine the distribution normality of continuous variables, which are expressed as the median (interquartile range). Depending on the normality of the variable distribution, the Student's t-test or Wilcoxon rank-sum test was conducted. We performed the Fisher's exact test or χ^2^-test to compare the categorical variables, which are expressed as percentages of numbers. Multivariable logistic regression models were conducted on urinary biomarkers that showed statistical significance in primary and secondary outcome, following initial univariable logistic regression analyses to identify potential predictors. Clinically relevant variables, obtainable from initial examination or initial laboratory findings in the emergency department, were included. Variables including SOFA scores were excluded due to the 24-h acquisition time limitation. To address potential multicollinearity in the multivariable logistic regression models, we calculated Variance Inflation Factor (VIF) values, and variables with a VIF >10 were excluded from the model to avoid distortion of the regression estimates. The results were expressed as odds ratios (OR) with corresponding 95 % confidence interval (CI) to indicate the strength and precision of the associations. To evaluate the added predictive value of the urinary biomarkers, we compared models with and without these biomarkers. The discriminatory power of each model was quantified using the are under the receiver operating characteristic curve (AUROC). DeLong's test was employed to statistically compare the AUROC values of the two models, determining whether the inclusion of urinary biomarkers significantly improved predictive accuracy. The DeLong's test, a non-parametric method, was used to compare the AUCs of the models due to its robustness and lack of distributional assumptions. This method is widely used in statistical and medical research to evaluate the discriminatory performance of predictive models. All data processing and statistical analyses were performed using R package software (version 4.2.1; R Foundation for Statistical Computing, Vienna, Austria). Statistical significance was defined as a two-tailed *p*-value <0.05.

## 3 Result

**Fig. 1:**
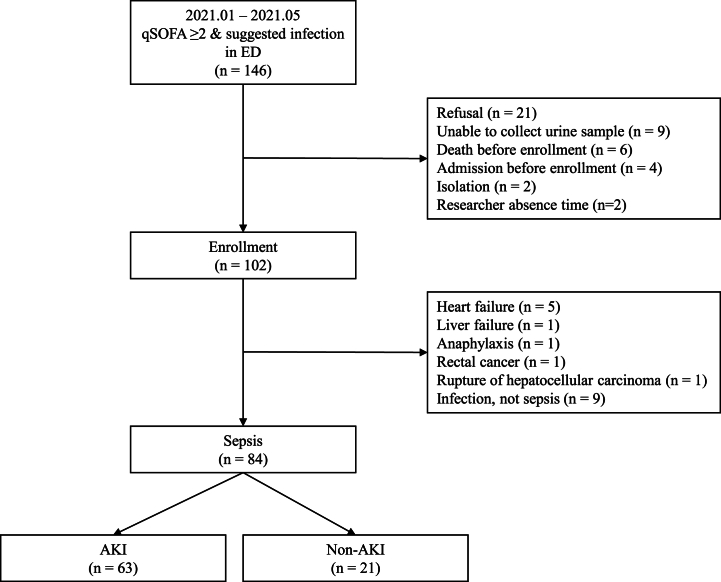
Flow chart of study population.

We enrolled 102 patients. Of them, 18 did not meet the diagnostic criteria for sepsis and were excluded. Finally, 84 patients were included in the analysis (Fig. 1). Among the enrolled patients, 16 (19.0 %) died within 30-day period, and 63 (75.0 %) were diagnosed with AKI.

Table 1 presents the baseline characteristics of patients according to the occurrence of AKI and mortality. Male was significantly more common in the non-AKI group compared to the AKI group (81 % vs. 53.9 %, p = 0.030). Patients who developed AKI were older (72.5 years vs. 67.0 years, p = 0.044) and had higher levels of blood urea nitrogen, creatinine, and C-reactive protein. The SOFA score was significantly higher in the AKI group (7.0 vs. 4.0, p = 0.001). The analysis excluding renal components of the SOFA score also showed higher scores in the AKI group (6.0 vs. 4.0, p = 0.012). Survivors were generally younger (71.5 years vs. 79.0 years, p = 0.042) and had higher albumin levels compared to non-survivors (3.2 g/dL vs. 2.5 g/dL, p = 0.025). Non-survivors had a higher SOFA score compared to survivors (8.5 vs. 6.0, p = 0.075).

**Table 1:** Baseline characteristics according to acute kidney injury occurrence and mortality.

	Total (n = 84)	AKI (n = 63)	Non-AKI (n = 21)	*p*-value	Survivor (n = 68)	Non-survivor (n = 16)	*p*-value
Sex (Male)	49 (58.3 %)	32 (50.8 %)	17 (81.0 %)	0.030	42 (61.8 %)	7 (43.8 %)	0.301
Age	72.5 [65.0–79.0]	76.0 [67.0–80.0]	67.0 [64.0–73.0]	0.044	71.5 [64.0–78.5]	79.0 [73.0–81.0]	0.042
Comorbidity							
Diabetes	25 (29.8 %)	16 (25.4 %)	9 (42.9 %)	0.215	22 (32.4 %)	3 (18.8 %)	0.443
Hypertension	45 (53.6 %)	33 (52.4 %)	12 (57.1 %)	0.899	35 (51.5 %)	10 (62.5 %)	0.605
Chronic liver disease	7 (8.3 %)	3 (4.8 %)	4 (19.0 %)	0.111	6 (8.8 %)	1 (6.2 %)	1.000
Heart failure	21 (25.0 %)	15 (23.8 %)	6 (28.6 %)	0.884	17 (25.0 %)	4 (25.0 %)	1.000
Chronic renal failure	8 (9.5 %)	7 (11.1 %)	1 (4.8 %)	0.668	5 (7.4 %)	3 (18.8 %)	0.355
Malignancy	40 (47.6 %)	26 (41.3 %)	14 (66.7 %)	0.077	31 (45.6 %)	9 (56.2 %)	0.624
Infection site				0.187			0.413
Genitourinary	20 (23.8 %)	16 (25.4 %)	4 (19.0 %)		17 (25.0 %)	3 (18.8 %)	
Intra-abdominal	30 (35.7 %)	22 (34.9 %)	8 (38.1 %)		24 (35.3 %)	6 (37.5 %)	
Pulmonary	16 (19.0 %)	14 (22.2 %)	2 (9.5 %)		11 (16.2 %)	5 (31.2 %)	
Other	9 (10.7 %)	7 (11.1 %)	2 (9.5 %)		9 (13.2 %)	0 (0.0 %)	
Unidentified	9 (10.7 %)	4 (6.3 %)	5 (23.8 %)		7 (10.3 %)	2 (12.5 %)	
Laboratory finding							
Hemoglobin (g/dL)	11.1 [9.6–12.7]	11.2 [9.6–12.9]	10.6 [9.0–11.8]	0.280	11.2 [9.6–12.7]	10.2 [7.7–12.7]	0.374
White blood cell ( × 10ˆ3/μL)	12.3 [8.2–18.4]	12.7 [9.7–20.1]	9.0 [7.3–15.3]	0.074	12.3 [8.0–18.4]	12.7 [8.6–22.6]	0.604
Platelet ( × 10ˆ3/μL)	164.0 [122.5–234.5]	159.0 [112.0–227.0]	186.0 [131.0–306.0]	0.339	156.5 [120.5–231.5]	183.5 [148.0–243.5]	0.327
Blood urea nitrogen (mg/dL)	32.5 [21.0–47.5]	39.0 [26.0–57.0]	19.0 [17.0–22.0]	0.000	29.0 [20.0–45.0]	53.0 [30.0–95.0]	0.004
Creatinine (mg/dL)	1.4 [0.9–2.0]	1.5 [1.2–2.3]	0.9 [0.8–1.2]	0.000	1.3 [0.9–1.8]	1.6 [1.0–3.3]	0.300
Total bilirubin (mg/dL)	1.4 [0.7–2.4]	1.4 [0.7–2.6]	1.2 [0.8–1.8]	0.893	1.3 [0.8–2.1]	1.4 [0.5–5.8]	0.780
Albumin (g/dL)	3.2 [2.6–3.7]	3.1 [2.5–3.6]	3.3 [2.8–3.7]	0.396	3.2 [2.8–3.7]	2.5 [2.2–3.3]	0.025
C-reactive protein (mg/dL)	13.6 [8.3–22.5]	17.3 [8.8–23.8]	10.4 [5.2–16.3]	0.039	13.6 [8.3–23.7]	12.7 [8.1–19.2]	0.624
Lactate (mmol/L)	2.2 [1.4–3.2]	2.2 [1.4–3.6]	2.3 [1.5–2.7]	0.958	2.0 [1.4–2.7]	2.8 [1.9–5.6]	0.106
Fractional excretion of sodium (%)	0.4 [0.2–0.7]	0.4 [0.2–0.9]	0.4 [0.2–0.6]	0.630	0.5 [0.2–0.9]	0.4 [0.2–0.6]	0.390
Urine Osmolality (mOsm/kg)	433.0 [313.0–553.0]	433.0 [313.0–518.0]	457.5 [320.0–573.0]	0.730	437.0 [313.0–553.0]	385.5 [321.0–530.0]	0.900
SOFA score							
Alla)	6.0 [4.0–9.0]	7.0 [5.0–9.5]	4.0 [3.0–6.0]	0.001	6.0 [3.5–8.0]	8.5 [5.0–11.5]	0.075
Except renalb)	5.0 [3.0–7.0]	6.0 [4.0–9.0]	4.0 [1.0–6.0]	0.012	5.0 [3.0–7.0]	6.0 [5.0–10.0]	0.071
Ventilator	4 (4.8 %)	4 (6.3 %)	0 (0.0 %)	0.554	3 (4.4 %)	1 (6.2 %)	1.000
Vasopressor	58 (69.0 %)	44 (69.8 %)	14 (66.7 %)	1.000	50 (73.5 %)	8 (50.0 %)	0.126

Data are expressed as N (%) or median [interquartile range], as appropriate.

AKI, acute kidney injury; SOFA, Sequential Organ Failure Assessment Score.

a The value calculated with all components of the SOFA score included.

b The SOFA score excluding the creatinine and urine output components.

Table 2 compares urinary biomarker concentrations between the patients diagnosed with (n = 63) and without AKI (n = 21) and between 30-day survivors (n = 68) and non-survivors (n = 16). NGAL and TIMP-2 × IGFBP-7 exhibited higher concentrations in patients with AKI than in those without AKI (364.4 vs 45.9 ng/mg, p = 0.014; 6.6 vs 2.9 ([ng/mL]^2^), p = 0.027). In the comparison between survivors and non-survivors, none of biomarkers demonstrated a statistically significant difference.

**Table 2:** Concentration of urine biomarkers according to acute kidney injury occurrence and mortality.

	AKI (n = 63)	No AKI (n = 21)	*p*-value	Survivor (n = 68)	Non-survivor (n = 16)	*p*-value
DKK3/uCr (pg/mg)	11554.3 [3473.6–31365.4]	3916.4 [2285.2–23860.7]	0.316	11111.9 [2743.5–35688.8]	6808.2 [1678.9–23121.9]	0.336
sTREM-1/uCr (pg/mg)	101.2 [33.4–442.9]	48.4 [21.1–242.8]	0.169	99.8 [31.8–389.0]	52.0 [5.2–136.8]	0.105
KIM-1/uCr (ng/mg)	4.3 [2.5–7.6]	3.7 [1.9–5.2]	0.186	4.2 [2.2–7.4]	3.6 [1.2–7.4]	0.390
NGAL/uCr (ng/mg)	364.4 [78.7–1351.9]	45.9 [16.2–443.2]	0.014	225.0 [39.0–924.7]	397.4 [71.8–1617.0]	0.422
TIMP-2/uCr (ng/mg)	10.4 [7.4–17.5]	9.1 [4.6–13.8]	0.076	9.1 [5.9–16.2]	12.5 [8.5–18.9]	0.143
IGFBP-7/uCr (pg/mg)	780.7 [372.2–1524.2]	511.9 [344.3–926.3]	0.086	669.2 [366.2–1403.0]	825.0 [224.8–1369.7]	0.754
TIMP-2 X IGFBP-7 ([ng/mL]^2^)	6.6 [1.9–40.7]	2.9 [0.9–3.7]	0.023	3.7 [1.8–17.5]	4.5 [1.8–69.0]	0.737

Data are expressed median [interquartile range].

AKI, acute kidney injury; DKK3, dickkopf-3; TREM-1, soluble triggering receptor expressed on myeloid cells-1; KIM-1, kidney injury molecule-1; NGAL, neutrophil gelatinase-associated lipocalin; TIMP-2, tissue inhibitor of metalloproteinases-2; IGFBP-7, insulin-like growth factor binding protein-7; uCr, Urine creatinine.

Table 3 presents a multivariable logistic regression analysis evaluating the association of urinary biomarkers with AKI in sepsis patients. Female is significantly associated with an increased risk of AKI, with ORs of 6.2 (95 % CI: 1.56–31.31, p = 0.015) and 6.66 (95 % CI: 1.65–33.84, p = 0.012) in the models with NGAL and TIMP-2 x IGFBP-7, respectively. Elevated creatinine levels also show a strong association with AKI, with ORs of 13.2 (95 % CI: 2.90–103.6, p = 0.004) and 8.88 (95 % CI: 2.16–66.29, p = 0.011) in the respective models. NGAL does not significantly predict AKI (OR: 1.0, 95 % CI: 0.93–1.10, p = 0.394), while TIMP-2 x IGFBP-7 shows a borderline association (OR: 1.09, 95 % CI: 1.00–1.10, p = 0.143). The AUC for models including these biomarkers (AUC with NGAL: 0.862, AUC with TIMP-2 x IGFBP-7: 0.839) is slightly higher than models without them (AUC without NGAL: 0.853, AUC without TIMP-2 x IGFBP-7: 0.853). The AUC improvement with NGAL did not show statistical significance (p = 0.417), whereas the TIMP-2 x IGFBP-7 was statistically significant (p = 0.018).

**Table 3:** Multivariable logistic regression analysis for the occurrence of acute kidney injury.

	Odds ratio	95 % CI	*p*-value		Odds Ratio	95 % CI	*p*-value
Sex (Female)	6.2	1.56-31.31	0.015	Sex (Female)	6.66	1.65-33.84	0.012
Age	1.0	0.98-1.08	0.216	Age	1.02	0.98-1.07	0.332
Creatinine (mg/dL)	13.2	2.90–100.63	0.004	Creatinine (mg/dL)	8.88	2.16–66.29	0.014
C-reactive protein (mg/dL)	1.0	0.93-1.10	0.751	C-reactive protein (mg/dL)	1.00	0.92-1.08	0.971
NGAL/uCr (ng/mg)	1.0	1.00–1.00	0.394	([ng/mL]^2^)	1.03	1.00–1.10	0.143
AUC with NGAL	0.862	0.762-0.862		AUC with TIMP-2 X IGFBP-7	0.889	0.800-0.899	
AUC without NGAL	0.853	0.754-0.853		AUC without TIMP-2 X IGFBP-7	0.853	0.754-0.853	
*p*-value∗			0.417	*p*-value∗			0.018

CI, confidence interval; NGAL, neutrophil gelatinase-associated lipocalin; AUC, area under the curve; TIMP-2, tissue inhibitor of metalloproteinases-2; IGFBP-7, insulin-like growth factor binding protein-7.

∗ DeLong's test.

Table 4, Table 5 display the concentration of urinary biomarkers according to the occurrence of AKI within 48 h in patients with initially normal creatinine (<1.2 mg/dL) and the results of the multiple logistic regression analysis for TIMP-2 × IGFBP-7. While all urinary biomarkers tended to be higher in patients who developed AKI, only IGFBP-7 and TIMP-2 × IGFBP-7 showed statistical significance. The multiple logistic regression analysis, similar to the results for the entire cohort, showed that female sex and elevated creatinine were associated with AKI. The AUC including TIMP-2 × IGFBP-7 was higher than the AUC without it (0.960 vs. 0.929) and showed statistical significance (p = 0.002).

**Table 4:** Concentration of urine biomarkers according to acute kidney injury occurrence and mortality in patients with initially normal creatinine.

	AKI (n = 49)	No AKI (n = 16)	*p*-value
DKK3/uCr (pg/mg)	10410.6 [2874.8–28005.0]	3477.5 [1831.4–35815.0]	0.561
sTREM-1/uCr (pg/mg)	98.3 [33.9–313.0]	84.8 [27.8–344.8]	0.709
KIM-1/uCr (ng/mg)	4.6 [2.7–7.7]	3.9 [2.1–5.7]	0.315
NGAL/uCr (ng/mg)	272.7 [72.3–846.7]	84.2 [16.1–1252.7]	0.151
TIMP-2/uCr (ng/mg)	10.4 [7.5–17.6]	9.1 [5.0–14.2]	0.195
IGFBP-7/uCr (pg/mg)	933.5 [537.8–1564.8]	491.5 [351.6–1090.1]	0.034
TIMP-2 X IGFBP-7 ([ng/mL]^2^)	8.4 [2.2–46.0]	2.5 [0.9–3.5]	0.002

Data are expressed median [interquartile range].

AKI, acute kidney injury; DKK3, dickkopf-3; TREM-1, soluble triggering receptor expressed on myeloid cells-1; KIM-1, kidney injury molecule-1; NGAL, neutrophil gelatinase-associated lipocalin; TIMP-2, tissue inhibitor of metalloproteinases-2; IGFBP-7, insulin-like growth factor binding protein-7; uCr, Urine creatinine.

**Table 5:** Multivariable logistic regression analysis for the occurrence of acute kidney injury in patients with initially normal creatinine.

	Odds Ratio	95 % CI	*p*-value
Sex (Female)	12.4	1.7–175.0	0.027
Age	1.1	1.0–1.2	0.128
Creatinine (mg/dL)	455.3	10.1–137339.2	0.008
C-reactive protein (mg/dL)	1.0	0.8-1.1	0.952
([ng/mL]^2^)	1.4	1.0–2.4	0.094
AUC with TIMP-2 X IGFBP-7	0.960	0.905-0.960	
AUC without TIMP-2 X IGFBP-7	0.929	0.863-0.929	
*p*-value∗			0.002

CI, confidence interval; AUC, area under the curve; TIMP-2, tissue inhibitor of metalloproteinases-2; IGFBP-7, insulin-like growth factor binding protein-7.

∗ DeLong's test.

## 4 Discussion

In this study, we evaluated the correlations between various urinary biomarkers and AKI diagnosis and 30-day survival in patients with sepsis who presented to the ED. No urinary damage biomarkers, including DKK-3, sTREM-1, KIM-1, NGAL, TIMP-2, IGFBP-7, and TIMP-2 × IGFBP-7, distinguished the 30-day survival. While NGAL and TIMP-2 × IGFBP-7 levels were significantly different between the AKI and non-AKI groups in unadjusted comparisons, these biomarkers did not demonstrate significant predictive value for AKI development in multivariable logistic regression analysis. However, when combined with plasma creatinine, TIMP-2 × IGFBP-7 showed potential to improve AKI prediction in sepsis patients presenting to the emergency department, as indicated by a significant result in the DeLong's test.

Urine TIMP-2 and IGFBP-7 are potential damage biomarkers of kidney injury associated with the G1 cell cycle arrest during the early phase of cell injury [[25], [26], [27], [28]]. This biomarker combination (TIMP-2 × IGFBP-7) is the only Food and Drug Administration-approved product for use in risk assessment for AKI in critically ill patients in the United States (NephroCheck) [29]. In a previous study from the PROCESS trial, which included patients with sepsis visiting the ED, there was no difference in 30-day survival between above and below TIMP-2 × IGFBP-7 levels (cut-off >2.0 ([ng/mL]^2^/1000) in the absence of functional criteria for AKI [18]. However, researchers identified an association between TIMP-2 × IGFBP-7 levels and 30-day survival upon dividing the AKI stages. Despite similar 30-day mortality (19.0 vs 19.3 %), our study demonstrated a higher incidence of AKI (75 % vs. 62.7 %), higher median age (72.5 vs 61 years), and greater proportion of underlying malignancies (47.6 vs 17.9 %). Furthermore, 70 % of patients surpassed the cut-off value (>2.0) in our study. By contrast, only 20 % of patients exceeded the threshold previously [18]. The difference in sampling time (initial urine in the ED vs. 6 h after enrollment) and potential variations in fluid treatment, which could have affected the urine concentration, provide possible explanations for these differences. Another multicenter study conducted in ED included approximately half of the patients with sepsis; urinary TIMP-2 × IGFBP-7 improved the predictive power for both AKI and 30-day mortality [30]. In contrast to the heterogeneous population that included all patients in ED with a relatively low AKI incidence (11.2 %), our study focused on a homogeneous sepsis population with a high AKI incidence (75 %). Therefore, urinary TIMP-2 × IGFBP-7 examination may offer additional benefits regarding short-term mortality in the general population of patients visiting the ED, in contrast to patients with sepsis and a high incidence of AKI.

NGAL is released from the kidney tubular cells under stress and neutrophils during inflammation; it has been extensively studied as a biomarker for AKI. In a post hoc subgroup analysis of patients with sepsis in the FINNAKI study [31], urine NGAL alone exhibited low discriminative power for AKI, RRT, and 90-day mortality. However, upon incorporation into the clinical risk model, the levels significantly improved AKI prediction and RRT, but not 90-day mortality. Consistent with the previous study, urine NGAL alone as well as other urinary biomarkers presented low discrimination power (0.6–0.7) for diagnosing AKI in patients with sepsis, thereby limiting its standalone utility. Unlike the previous study, urine NGAL failed to add discriminative power to the multivariable model, compared with TIMP-2 × IGFBP-7. However, the magnitude of improvements in AKI prediction was not clinically meaningful, indicating low discriminatory power, consistent with previous findings using decision curve analysis [31].

In our study, urinary damage biomarkers, including DKK-3, sTREM-1, and KIM-1, could not distinguish patients with AKI or predict the 30-day survival. In a pediatric ICU cohort comprising patients with sepsis, urinary DKK3 levels upon ICU admission were independently associated with an increased risk of AKI, septic AKI, and ICU mortality [14]. In an adult ICU study comprising patients with sepsis, urinary sTREM-1 was identified as a sensitive biomarker for diagnosing septic AKI and predicting prognosis [15]. Furthermore, higher concentrations of urinary KIM-1 at 24 and 48 h have been observed in non-survivors [16]. These studies associated higher levels of urinary DKK-3, sTREM-1, and KIM-1 with poor outcomes; by contrast, we did not observe any association between these biomarkers and the 30-day survival. Interestingly, these biomarkers tended to be lower in non-survivors, despite statistical insignificance. This discrepancy may be attributed to the heterogeneous cohort within the pediatric population, ICU setting, delayed urine sampling time, and fluid treatment. Notably, we focused on the clinical utility of these biomarkers in initial urine samples of patients with sepsis presenting at the ED. Among biomarkers, TIMP-2 × IGFBP-7 levels in the initial urine showed possibility in the ED patient cohort [32].

This study has several limitations. First, the population was relatively small and the patients were enrolled from a single department, which may have limited the power and generalizability of our findings. Furthermore, the wide confidence interval observed for creatinine in the multivariable analysis highlights the need for larger datasets or additional cohorts to improve the precision of the estimate. Second, the lack of longitudinal collection of urine samples prevents gathering serial information on the biomarkers, although the feasibility of utilizing a single urine sample at the initial ED presentation for minimizing fluctuations. Third, RRT application underwent limited analysis because only two patients received treatment in the first study. Fourth, study was conducted based on clinical practice guidelines of surviving sepsis campaign from 2017 [33]. Although many of recommendations, specifically those related to the diagnosis of AKI, remain unchanged in 2021 compared to 2017 ^5^, there might be influences due to the evolution of management practices.

In summary, we did not find significant associations between initial urinary biomarkers and AKI diagnosis or 30-day mortality in patients with sepsis presenting to the emergency department. Although NGAL and TIMP-2 × IGFBP-7 were significantly different between the AKI and non-AKI groups in unadjusted analysis, multivariable logistic regression did not confirm these biomarkers as predictors. However, the inclusion of urinary TIMP-2 × IGFBP-7 improved the performance of model for predicting AKI. Further multicenter studies with larger populations are needed to confirm its clinical utility and explore its role.

## CRediT authorship contribution statement

**Sumin Baek:** Writing – original draft, Methodology, Formal analysis, Data curation, Conceptualization. **Inwon Park:** Writing – review & editing, Writing – original draft, Methodology, Formal analysis, Data curation, Conceptualization. **Seonghye Kim:** Writing – original draft, Investigation. **Young Woo Um:** Writing – original draft, Investigation. **Hee Eun Kim:** Writing – original draft, Investigation. **Kyunghoon Lee:** Writing – review & editing, Investigation. **Jae Hyuk Lee:** Writing – review & editing, Supervision, Conceptualization. **You Hwan Jo:** Writing – review & editing, Supervision, Conceptualization.

## Ethics statement

The study design and protocol were approved by the Institutional Review Board of Seoul National University Bundang Hospital (IRB No.B-2011-648-304). Informed consent was obtained from all patients or from their legal guardians.

## Data availability statement

The data supporting the findings of this study are included in the article. Additional data are available upon request from the corresponding author.

## Funding

This work was supported by the Research fund from the Department of Emergency Medicine inSeoul 10.13039/100018366National University 10.13039/100008212College of Medicine (No. 800–20200522), from the 10.13039/100016275SNUBH research fund (No. 02-2021-00018), 10.13039/501100003725National Research Foundation of Korea (NRF) grants funded by the Korea government (10.13039/501100014188MSIT) (NRF-2021R1C1C1003214), and Korea 10.13039/100018696Health Technology R&D Project through the 10.13039/501100003710Korea Health Industry Development Institute (10.13039/501100003710KHIDI), funded by the 10.13039/100009647Ministry of Health & Welfare, Republic of Korea (RS-2024-00439462).

## Declaration of competing interest

The authors declare the following financial interests/personal relationships which may be considered as potential competing interests: Inwon Park reports financial support was provided by 10.13039/501100003725National Research Foundation (NRF) of Korea. Inwon Park reports financial support was provided by Korea Health Industry Development Institute (KHIDI). Inwon Park reports financial support was provided by Seoul 10.13039/100018366National University 10.13039/100008212College of Medicine. Inwon Park reports financial support was provided by Seoul 10.13039/100018366National University Bundang Hospital. If there are other authors, they declare that they have no known competing financial interests or personal relationships that could have appeared to influence the work reported in this paper.

## References

[1] Singer M., Deutschman C.S., Seymour C.W., Shankar-Hari M., Annane D., Bauer M. (2016). The third international consensus definitions for sepsis and septic shock (Sepsis-3). JAMA.

[2] Kim J., Kim K., Lee H., Ahn S. (2019). Epidemiology of sepsis in Korea: a population-based study of incidence, mortality, cost and risk factors for death in sepsis. Clin Exp Emerg Med.

[3] Zarbock A., Nadim M.K., Pickkers P., Gomez H., Bell S., Joannidis M. (2023). Sepsis-associated acute kidney injury: consensus report of the 28th Acute Disease Quality Initiative workgroup. Nat. Rev. Nephrol..

[4] Peerapornratana S., Manrique-Caballero C.L., Gomez H., Kellum J.A. (2019). Acute kidney injury from sepsis: current concepts, epidemiology, pathophysiology, prevention and treatment. Kidney Int..

[5] Evans L., Rhodes A., Alhazzani W., Antonelli M., Coopersmith C.M., French C. (2021). Surviving sepsis campaign: international guidelines for management of sepsis and septic shock 2021. Crit. Care Med..

[6] Poston J.T., Koyner J.L. (2019). Sepsis associated acute kidney injury. BMJ.

[7] Suh G.J., Shin T.G., Kwon W.Y., Kim K., Jo Y.H., Choi S.H. (2023). Hemodynamic management of septic shock: beyond the Surviving Sepsis Campaign guidelines. Clin Exp Emerg Med.

[8] Oberlin M., Balen F., Bertrand L., Chapeau N., San Cirilo B., Ruols E. (2020). Sepsis prevalence among patients with suspected infection in emergency department: a multicenter prospective cohort study. Eur. J. Emerg. Med..

[9] Kellum J.A., Lameire N., Group K.A.G.W. (2013). Diagnosis, evaluation, and management of acute kidney injury: a KDIGO summary (Part 1). Crit. Care.

[10] Martinez D.A., Levin S.R., Klein E.Y., Parikh C.R., Menez S., Taylor R.A. (2020). Early prediction of acute kidney injury in the emergency department with machine-learning methods applied to electronic Health record data. Ann. Emerg. Med..

[11] Bouchard J. (2021). Estimating baseline serum creatinine for assessing acute kidney injury: not a one size fits all approach. Kidney Int Rep.

[12] Endre Z.H., Pickering J.W., Walker R.J. (2011). Clearance and beyond: the complementary roles of GFR measurement and injury biomarkers in acute kidney injury (AKI). Am. J. Physiol. Ren. Physiol..

[13] Waikar S.S., Bonventre J.V. (2009). Creatinine kinetics and the definition of acute kidney injury. J. Am. Soc. Nephrol..

[14] Hu J., Zhou Y., Huang H., Kuai Y., Chen J., Bai Z. (2023). Prediction of urinary dickkopf-3 for AKI, sepsis-associated AKI, and PICU mortality in children. Pediatr. Res..

[15] Su L.X., Feng L., Zhang J., Xiao Y.J., Jia Y.H., Yan P. (2011). Diagnostic value of urine sTREM-1 for sepsis and relevant acute kidney injuries: a prospective study. Crit. Care.

[16] Tu Y., Wang H., Sun R., Ni Y., Ma L., Xv F. (2014). Urinary netrin-1 and KIM-1 as early biomarkers for septic acute kidney injury. Ren. Fail..

[17] Zhang A., Cai Y., Wang P.F., Qu J.N., Luo Z.C., Chen X.D. (2016). Diagnosis and prognosis of neutrophil gelatinase-associated lipocalin for acute kidney injury with sepsis: a systematic review and meta-analysis. Crit. Care.

[18] Molinari L., Del Rio-Pertuz G., Smith A., Landsittel D.P., Singbartl K., Palevsky P.M. (2022). Utility of biomarkers for sepsis-associated acute kidney injury staging. JAMA Netw. Open.

[19] Hsu Y.C., Hsu C.W. (2019). Septic acute kidney injury patients in emergency department: the risk factors and its correlation to serum lactate. Am. J. Emerg. Med..

[20] Turgman O., Schinkel M., Wiersinga W.J. (2023). Host response biomarkers for sepsis in the emergency room. Crit. Care.

[21] Nickolas T.L., O'Rourke M.J., Yang J., Sise M.E., Canetta P.A., Barasch N. (2008). Sensitivity and specificity of a single emergency department measurement of urinary neutrophil gelatinase-associated lipocalin for diagnosing acute kidney injury. Ann. Intern. Med..

[22] Mishra J., Dent C., Tarabishi R., Mitsnefes M.M., Ma Q., Kelly C. (2005). Neutrophil gelatinase-associated lipocalin (NGAL) as a biomarker for acute renal injury after cardiac surgery. Lancet.

[23] Siew E.D., Matheny M.E. (2015). Choice of reference serum creatinine in defining acute kidney injury. Nephron.

[24] Graversen H.V., Jensen S.K., Vestergaard S.V., Heide-Jorgensen U., Christiansen C.F. (2022). Defining baseline creatinine for identification of AKI in population-based laboratory databases: a Danish nationwide cohort study. Kidney.

[25] Emlet D.R., Pastor-Soler N., Marciszyn A., Wen X., Gomez H., Wht Humphries (2017). Insulin-like growth factor binding protein 7 and tissue inhibitor of metalloproteinases-2: differential expression and secretion in human kidney tubule cells. Am. J. Physiol. Ren. Physiol..

[26] Kashani K., Al-Khafaji A., Ardiles T., Artigas A., Bagshaw S.M., Bell M. (2013). Discovery and validation of cell cycle arrest biomarkers in human acute kidney injury. Crit. Care.

[27] Bihorac A., Chawla L.S., Shaw A.D., Al-Khafaji A., Davison D.L., Demuth G.E. (2014). Validation of cell-cycle arrest biomarkers for acute kidney injury using clinical adjudication. Am. J. Respir. Crit. Care Med..

[28] Honore P.M., Nguyen H.B., Gong M., Chawla L.S., Bagshaw S.M., Artigas A. (2016). Urinary tissue inhibitor of metalloproteinase-2 and insulin-like growth factor-binding protein 7 for risk stratification of acute kidney injury in patients with sepsis. Crit. Care Med..

[29] Erstad B.L. (2022). Usefulness of the biomarker TIMP-2∗igfbp7 for acute kidney injury assessment in critically ill patients: a narrative review. Ann. Pharmacother..

[30] Yang H.S., Hur M., Lee K.R., Kim H., Kim H.Y., Kim J.W. (2022). Biomarker rule-in or rule-out in patients with acute diseases for validation of acute kidney injury in the emergency department (brava): a multicenter study evaluating urinary TIMP-2/IGFBP7. Ann Lab Med.

[31] Tornblom S., Nisula S., Petaja L., Vaara S.T., Haapio M., Pesonen E. (2020). Urine NGAL as a biomarker for septic AKI: a critical appraisal of clinical utility-data from the observational FINNAKI study. Ann. Intensive Care.

[32] Fan W., Ankawi G., Zhang J., Digvijay K., Giavarina D., Yin Y. (2019). Current understanding and future directions in the application of TIMP-2 and IGFBP7 in AKI clinical practice. Clin. Chem. Lab. Med..

[33] Rhodes A., Evans L.E., Alhazzani W., Levy M.M., Antonelli M., Ferrer R. (2017). Surviving sepsis campaign: international guidelines for management of sepsis and septic shock: 2016. Intensive Care Med..

